# Cell-free DNA in patients with sepsis: long term trajectory and association with 28-day mortality and sepsis-associated acute kidney injury

**DOI:** 10.3389/fimmu.2024.1382003

**Published:** 2024-05-13

**Authors:** Sophie Dennhardt, Iuliana-Andreea Ceanga, Philipp Baumbach, Mona Amiratashani, Sarah Kröller, Sina M. Coldewey

**Affiliations:** ^1^ Department of Anesthesiology and Intensive Care Medicine, Jena University Hospital, Friedrich Schiller University Jena, Jena, Germany; ^2^ Septomics Research Centre, Jena University Hospital, Friedrich Schiller University Jena, Jena, Germany; ^3^ Center for Sepsis Control and Care, Jena University Hospital, Jena, Germany

**Keywords:** acute kidney injury, cell-free DNA, intensive care unit, mitochondrial DNA, mortality, nuclear DNA, renal replacement therapy, sepsis

## Abstract

**Introduction:**

Outcome-prediction in patients with sepsis is challenging and currently relies on the serial measurement of many parameters. Standard diagnostic tools, such as serum creatinine (SCr), lack sensitivity and specificity for acute kidney injury (AKI). Circulating cell-free DNA (cfDNA), which can be obtained from liquid biopsies, can potentially contribute to the quantification of tissue damage and the prediction of sepsis mortality and sepsis-associated AKI (SA-AKI).

**Methods:**

We investigated the clinical significance of cfDNA levels as a predictor of 28-day mortality, the occurrence of SA-AKI and the initiation of renal replacement therapy (RRT) in patients with sepsis. Furthermore, we investigated the long-term course of cfDNA levels in sepsis survivors at 6 and 12 months after sepsis onset. Specifically, we measured mitochondrial DNA (mitochondrially encoded NADH-ubiquinone oxidoreductase chain 1, *mt-ND1*, and mitochondrially encoded cytochrome C oxidase subunit III, *mt-CO3*) and nuclear DNA (nuclear ribosomal protein S18, *n-Rps18*) in 81 healthy controls and all available samples of 150 intensive care unit patients with sepsis obtained at 3 ± 1 days, 7 ± 1 days, 6 ± 2 months and 12 ± 2 months after sepsis onset.

**Results:**

Our analysis revealed that, at day 3, patients with sepsis had elevated levels of cfDNA (*mt-ND1*, and *n-Rps18*, all p<0.001) which decreased after the acute phase of sepsis. 28-day non-survivors of sepsis (16%) had higher levels of cfDNA (all p<0.05) compared with 28-day survivors (84%). Patients with SA-AKI had higher levels of cfDNA compared to patients without AKI (all p<0.05). Cell-free DNA was also significantly increased in patients requiring RRT (all p<0.05). All parameters improved the AUC for SCr in predicting RRT (AUC=0.88) as well as APACHE II in predicting mortality (AUC=0.86).

**Conclusion:**

In summary, cfDNA could potentially improve risk prediction models for mortality, SA-AKI and RRT in patients with sepsis. The predictive value of cfDNA, even with a single measurement at the onset of sepsis, could offer a significant advantage over conventional diagnostic methods that require repeated measurements or a baseline value for risk assessment. Considering that our data show that cfDNA levels decrease after the first insult, future studies could investigate cfDNA as a “memoryless” marker and thus bring further innovation to the complex field of SA-AKI diagnostics.

## 1 Introduction

Patients with sepsis have a high mortality risk. By definition, they develop infection-related organ dysfunction, with sepsis-related acute kidney injury (SA-AKI) occurring particularly frequently. With an overall mortality estimated at around 27% which increases to 42% for patients with sepsis admitted to the intensive care unit (ICU) (1), sepsis remains a diagnostic and therapeutic challenge across the medical field. The care of sepsis patients is complex and requires intensive clinical and laboratory monitoring for early diagnosis of complications and organ damage. In clinical practice, this often translates into high frequency blood sample tests acquisition, as well as intensive monitoring of clinical parameters and calculation of clinical risk scores (such as APACHE II). These procedures, while currently the standard of care for patients with sepsis, have several drawbacks such as increased costs, time-consuming management, and proneness to errors (e. g. in automated APACHE II protocols). Furthermore, diagnosing sepsis-associated organ damage may be delayed due to a lack of baseline values for specific patients, as it may be, for example, the case in patients who develop SA-AKI. The presence of AKI has a severe impact on patient outcome and is associated with prolonged hospitalization and ICU stay, disability, poor quality of life and higher mortality (2, 3). Both conditions – sepsis and AKI – are mutually reinforcing and worsen each other’s prognosis (2, 4, 5). Despite its impact on patient outcomes, predicting the development of SA-AKI remains elusive, especially at onset and early stages. The diagnostic challenge arises from the lack of injury markers that directly relate to the degree of kidney damage, which can currently only be determined by tissue biopsy (6). The diagnostic markers serum creatinine (SCr) and urine output have both low sensitivity and specificity for AKI (7, 8), especially in the presence of sepsis. Therefore, the development and refinement of clinical methods to quantify organ dysfunction and disease severity in patients with sepsis is necessary and remains the subject of current research.

Liquid biopsies can be employed to quantify endogenous cell-free DNA (cfDNA), which is either released from dying cells during necrosis, apoptosis or actively secreted during netosis, as a special form of pathogen-induced cell death, which releases neutrophil extracellular traps (NETs) (9). While resembling an anti-viral host response strategy, NETs also contribute to a thrombo-inflammatory state (10). Via TLR9-receptor signaling, cfDNA can act as danger-associated molecular patterns (DAMPs) further exacerbating the inflammatory response (11). These innate immune response mechanisms are described as being triggers for AKI (12), but it remains debatable whether cfDNA is merely a marker of tissue-injury during sepsis and SA-AKI or has its own role in the pathophysiology of the condition (13). Cell-free DNA can be detected in different samples, such as blood, urine, saliva, as well as other body fluids (14–16). Cell-free DNA shows good potential for predicting outcomes and correlates, among others, with prognosis in patients with inflammation (17), myocardial infarction (18, 19), and stroke (20, 21). More recently, liquid biopsies have been correlated with the degree of tissue damage, hence being proposed as markers of inflammation, kidney dysfunction, morbidity and mortality in patients with sepsis. While some studies suggest that both nuclear DNA (nDNA) and mitochondrial DNA (mtDNA) have limited roles in sepsis (22), others reported that mtDNA might serve as a predictor for 28-day mortality in patients with sepsis originating from mixed infection sites (22–24). Cell-free DNA may also have the potential to predict kidney damage in SA-AKI, complications of SA-AKI, or mortality in patients with sepsis. However, its clinical role in patients with sepsis is currently unclear due to conflicting reports. The aims of our study were to investigate both the potential of cfDNA as a prognostic marker for 28-day mortality in patients with sepsis and its significance in predicting the need for renal replacement therapy (RRT) in patients with AKI as well as to present the dynamic of cfDNA from the acute phase of sepsis up to 12 months after sepsis onset.

## 2 Material and methods

### 2.1 Study design and study population

In the present study, cfDNA was analyzed in blood samples from patients with sepsis and healthy controls who participated in the prospective longitudinal ICROS study (“Identification of cardiovascular and molecular prognostic factors for the medium-term and long-term outcomes of sepsis”) (25). For a detailed description of this study, we refer to the published protocol (25). Briefly, patients with surgical and non-surgical sepsis admitted to the ICU of Jena University Hospital were enrolled between 24/05/2018 and 24/03/2021. Sepsis was diagnosed according to the Sepsis-3 criteria (26). Main exclusion criteria included significant heart, end-stage renal or hepatic disease, pregnancy or breastfeeding as well as therapeutic limitation or a do-not-resuscitate order. We excluded patients with COVID-19-associated sepsis from the analysis as they were not part of the original study protocol and COVID-19 patients were not within the scope of this analysis.

In the acute phase of sepsis, study visits took place 3 ± 1 days (T1) and 7 ± 1 days (T2) after sepsis onset. In addition, study visits took place in the early recovery phase (T3, ICU-discharge, ± 3 days) and late recovery phase 6 ± 2 months (T4) and12 ± 2 months (T5) after sepsis onset.

In addition, 81 healthy volunteers were recruited between 09/05/2018 and 16/06/2022. The study was conducted in accordance with the Declaration of Helsinki and approved by the Ethics Committee of the Friedrich-Schiller-University Jena (ClinicalTrials registration: NCT03620409). Every participant, legal representative or proxy gave their signed written informed consent or, if none of the situations applied, an independent medical doctor was consulted for preliminary consent (25).

### 2.2 Clinical data collection

Clinical data was gathered from the electronic patient data management system COPRA 6^©^, at the screening visit and the in-hospital visits (T1, T2). We also recorded data regarding the patients’ kidney function before and after admission to the ICU, the need for RRT during the first 28 days of ICU stay, 28-day mortality, as well as the Acute Physiology and Chronic Health Evaluation (APACHE) II score at T1. APACHE II was chosen because it is usually measured at ICU admission and does not need sequential measurement to predict negative outcomes. It is a complex assessment tool including 12 physiological parameters and the patient’s medical history and age.

### 2.3 Diagnosis of AKI and sepsis-associated AKI

AKI was diagnosed according to the current KDIGO criteria (27) based on one of the following: increase in SCr ≥ 0.3 mg/dL within 48 hours, or an increase in SCr ≥ 1.5 times from baseline (that is known or presumed to have occurred within the past 7 days), or urine volume < 0.5mL/kg/h for 6 hours. In order to diagnose AKI, baseline SCr was needed, a laboratory value which was not available for all patients. After careful consideration of the literature (28–31) and analyzing the particularities of our cohort, we decided to infer the missing information regarding baseline SCr by reviewing the patients’ electronic data over the last 365 days before ICU admission. In cases where patients had no history of kidney disease and a retrospective determination of the SCr was not possible (n=23 patients) the mean value of the laboratory reference interval for SCr was used as the baseline values. Since normal SCr levels vary by age and sex, reference ranges are adjusted (32) by the test manufacturer (Roche diagnostics) to take into account these two variables. Additionally, we have gathered daily information regarding the urinary output during the ICU stay up to 28 days. This allowed us to check the validity of our diagnosis and to potentially detect cases where an AKI diagnosis would be unlikely based on the SCr alone (e. g. patients with low nutritional scores, low muscle mass, malnourished patients). SA-AKI was defined based on the recommendation of the 28^th^ Acute Disease Quality Initiative workgroup, as AKI diagnosed in the presence of sepsis (2).

### 2.4 Blood sampling

Blood samples used for the present study were obtained on T1, T2, T4, and T5. At each time point, plasma was snap-frozen in liquid nitrogen and stored at −80°C for later DNA isolation and quantification. Cell-free DNA was not measured at ICU-discharge (T3) due to the high variability of this time point (ICU discharge ranging between 1 day and 108 days).

### 2.5 Isolation and quantification of cfDNA

Plasma obtained from EDTA-anticoagulated whole blood was centrifuged again at 3 000 g and 4°C to obtain platelet-poor plasma. To isolate cell- and microvesicle-free DNA only, the platelet-poor plasma was then filtered through a 0.2 µm PVDF membrane filter tube (Whatman). 200 µl of the filtered plasma were used for DNA isolation with the QiaAmp DNA kit (Qiagen). According to the producer’s manual, carrier DNA (poly dA:dT, Invivogen) was added to the isolation buffer to increase the isolation yield. Isolated DNA was quantified spectrophotometrically and diluted to 0.325 ng/µl for direct use in qPCR. Mitochondrial DNA quantification was performed by quantitative real-time PCR on a CFX96™ Touch Real-Time PCR Detection System (Bio-Rad Laboratories, Inc.) using SsoAdvanced™ Universal SYBR^®^ Green Supermix (Bio-Rad) and PrimePCR™ assays *mt-ND1* (NADH-ubiquinone oxidoreductase chain 1, qHsaCED0021828) and *mt-CO3* (cytochrome C oxidase subunit III, qHsaCED0048405; Bio-Rad Laboratories, Inc.). Nuclear DNA quantification was performed by quantitative real-time PCR on a CFX96™ Touch Real-Time PCR Detection System (Bio-Rad Laboratories, Inc.) using SsoAdvanced™ Universal Probes Supermix (Bio-Rad) and PrimePCR™ probe assay *n-Rps18* (ribosomal protein S18, qHsaCEP0040177; Bio-Rad Laboratories, Inc.). Copy numbers of *mt-ND1*, *mt-CO3* and *n-Rps18* were extrapolated from standard curves of serially diluted template DNA (2 million copies/µl stock concentration).

### 2.6 Primary and secondary aims and outcome measures

The primary aim was the evaluation of the prognostic relevance of cfDNA (values of *mt-ND1*, *mt-CO3*, *n-Rps18* at T1) for 28-day mortality. Secondary aims comprised the evaluation of the prognostic relevance of cfDNA (values of *mt-ND1*, *mt-CO3*, *n-Rps18* at T1) for RRT up to day 28 after sepsis onset. Further aims comprised the characterization of cfDNA in the acute phase of sepsis (T1, T2) and long-term recovery after sepsis (T4, T5). This included the group comparisons between patients with sepsis and healthy controls.

### 2.7 Statistical analysis

#### 2.7.1 Descriptive analysis

We report median and interquartile range (IQR; first to third quartile; Q_1_–Q_3_) for continuous variables. For categorical variables, we report absolute and relative frequencies (%). Due to the high range in variables of cfDNA, we performed log10-transformation. To account for zero values, 1 was added to each value.

#### 2.7.2 Statistical tests

For unpaired comparisons of metric variables, we applied Mann-Whitney *U* tests (two groups) or Kruskal-Wallis-test with Dunn’s *post hoc* tests (more than two groups). For paired metric data (long-term courses), Friedman tests with Dunn’s *post hoc* tests were applied. For unpaired categorical variables, we applied Fisher’s exact tests.

#### 2.7.3 Binary logistic regression models

**Twenty-eight-day mortality.** We tested 2 series of logistic regression models with 28-day mortality as dependent variable. In the first series, we evaluated the prognostic value of SCr and cfDNA variables. The models were built with three different biomarker combinations as independent variables: model A contained only SCr, model B contained *mt-ND1*, *mt-CO3* and *n-Rps18* and model C contained SCr, *mt-ND1*, *mt-CO3* and *n-Rps18*. In the second series, we followed the same approach, but instead of SCr, we modeled APACHE-II scores at T1 as an established score for mortality prediction.

**RRT (up to day 28).** For RRT as dependent variable, three different biomarker combinations as covariates were tested: model A contained only SCr, model B contained *mt-ND1*, *mt-CO3* and *n-Rps18* and model C contained SCr *mt-ND1*, *mt-CO3* and *n-Rps18*.

For all models, we report the odds ratio (OR) including 95% confidence interval (95% CI) and the p value. With a variance inflation factor (VIF) of less than 10, we have assumed an acceptable level of multicollinearity (**Supplementary Table 1**). Model based predicted probabilities were calculated for each patient and model and then analyzed using in receiver operating characteristic (ROC) curve analysis. We report values of sensitivity and specificity for the optimal cut-off based on Youden’s index. Finally, we tested the superiority of all models B and C compared to all models A using DeLong’s tests (one sided testing).

Data were analyzed with the CFX Manager™ 3.1 software (Bio-Rad Laboratories, Inc.), GraphPad Prism 7.05 for Windows (GraphPad Software, Inc., San Diego, CA, USA), IBM SPSS Statistics for Windows, Version 27.0 (Armonk, NY: IBM Corp) and R (Version 4.1.0) under R Studio (Version 1.4.1717). We considered p values <0.05 as statistically significant.

## 3 Results

**Figure 1:**
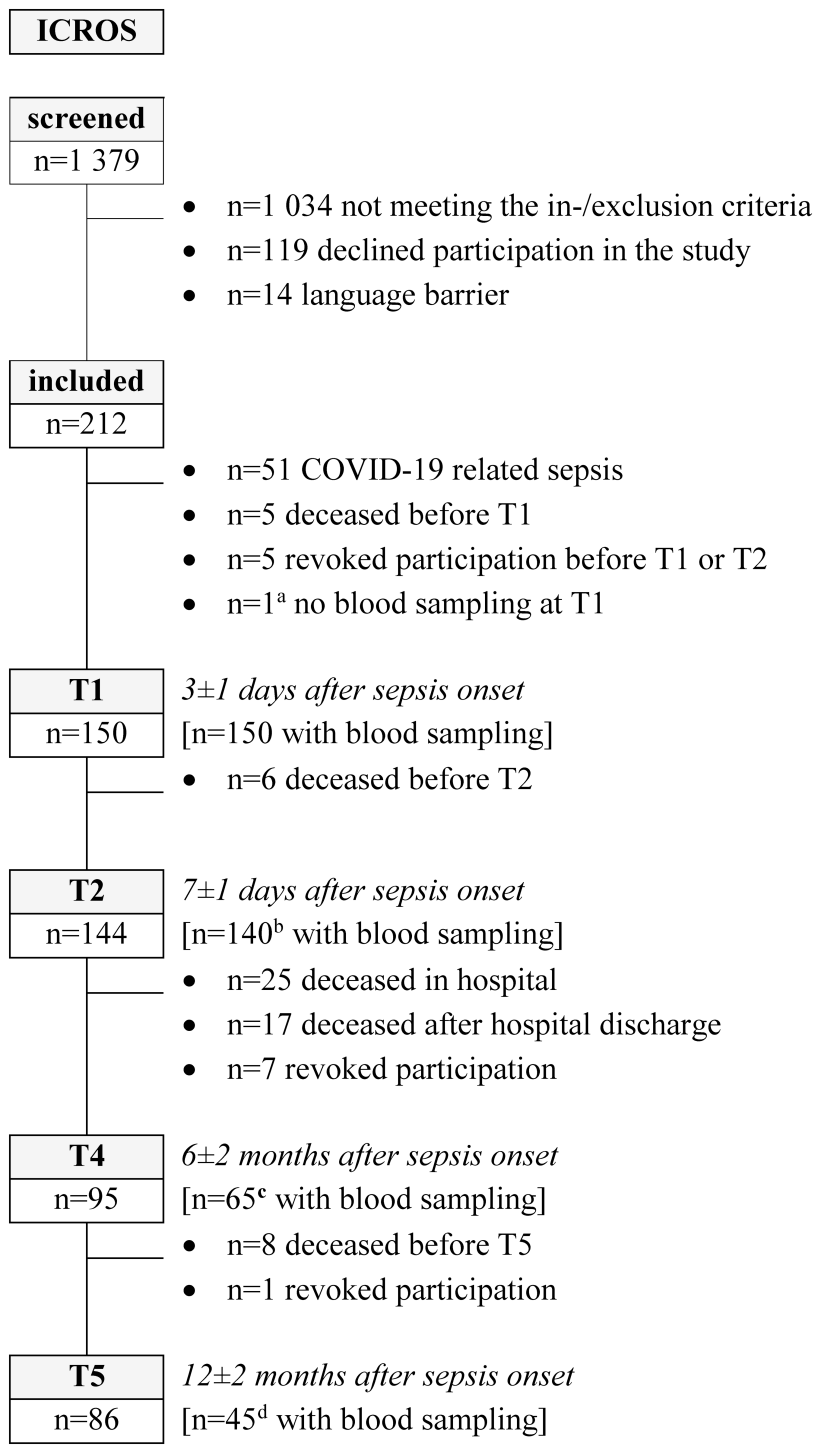
Patient flow chart. The study visit T3 (discharge from intensive care unit, ± 3 days) was not part of this analysis [^a^ excluded from analyses because not available for the primary endpoint; ^b^ refusal (n=2), no blood sampling (n=1) or already discharged from hospital (n=1); ^c^ refusal (n=6) or phone interview (n=24); ^d^ refusal (n=9), phone interview (n=26) or not sufficient plasma available to measure cell-free DNA (n=6)]. Further, 81 healthy controls were included in the ICROS-study and current analysis (ICROS, Identification of cardiovascular and molecular prognostic factors for the medium-term and long-term outcomes of sepsis).

The patient inclusion and number of patients with blood samples are summarized in **Figure 1**.

### 3.1 Demographic and clinical characteristics

Of the 150 patients with sepsis included in this analysis, 56 (37.3%) patients were female. The median age was 66 years (IQR: 56–75). We found no significant differences in age and sex between patients and healthy controls (median [IQR], age: 65 [53–73], p=0.404; sex: n=30/81, 37% females). Patients had a significantly higher body mass index (BMI, 27.8 [23.7–32.4], p<0.001) and Charlson comorbidity index (CCI, 2 [1–4], p<0.001) compared to healthy controls (BMI: 25.3 [23.0–27.2], CCI: 0 [0–0]). Further clinical characteristics of the patients are shown in **Table 1**.

**Table 1:** Patient characteristics.

Variable	Patients with sepsis (n=150)
Age (years)	66 (56–76)
Sex (female)	56 (37.3%)
Body Mass Index (kg/m²)	27.8 (23.7–32.4)
Charlson Comorbidity Index (points)	2 (1–4)
APACHE II at ICU admission (points)	23 (17–28)
SOFA at sepsis onset (points)	9 (7–11)
Sepsis focus	
Respiratory	72 (48%)
Wound/bone/soft tissue	19 (12.7%)
Intra-Abdominal	55 (36.7%)
Urogenital	19 (12.7%)
Primary bacteraemia	6 (4%)
Other	17 (11.4%)
Multiple foci	35 (23.3%)
Type of referral	
Surgical: emergency	55 (36.7%)
Surgical: elective	46 (30.7%)
Non-surgical emergency	49 (32.7%)
CKD stage	
None	89 (59.3%)
1	1 (0.7%)
2	28 (18.7%)
3	25 (16.6%)
4	6 (4%)
5	1 (0.7%)
AKI stage	
None	68 (45.3%)
1	12 (8%)
2	15 (10%)
3	55 (36.7%)
AKI on CKD (yes)	43 (28.7%)
RRT (yes)	44 (29.3%)
Discharge within 28 days (yes)	108 (72.0%)
28-day mortality (non-survivors)	24 (16.0%)
Length of stay: ICU (days)	10 (4–23)
Length of stay: hospital (days)	30 (20–47)

AKI, acute kidney injury: defined according to the KDIGO criteria: either an increase in serum creatinine (SCr) ≥ 0.3 mg/dL within 48 hours, or an increase in SCr ≥ 1.5 times from baseline, or urine volume < 0.5mL/kg/h for 6 hours; APACHE II, Acute Physiology and Chronic Health Evaluation II; CKD, chronic kidney disease; ICU, intensive care unit; RRT, renal replacement therapy; SOFA, Sequential Organ Failure Assessment.

Data are presented as median (interquartile range) or count (percentage).

Forty-one percent of patient**s** had preexisting chronic kidney disease (CKD), 54.7% were diagnosed with SA-AKI (either on admission or during the ICU stay) and 28.7% with AKI on CKD. Renal replacement therapy was needed in 29.3% of the patients. Twenty-four patients (16.0%) died within 28 days.

### 3.2 Levels of circulating mtDNA and nDNA are elevated in patients with sepsis

We first tested the hypothesis that cfDNA is increased in the acute phase of sepsis (T1), reflecting tissue injury. For this, we assessed the copy numbers of *mt-ND1, mt-CO3* and *n-Rps18* as surrogate parameters for cfDNA in plasma.

**Figure 2:**
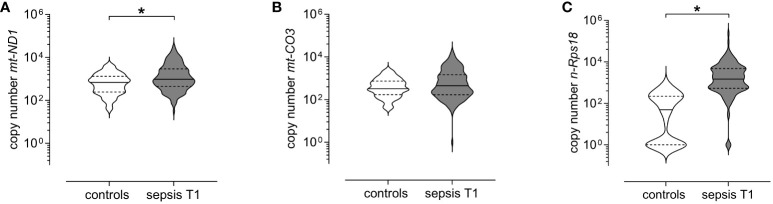
Levels of circulating mitochondrial DNA and nuclear DNA at T1 (3 ± 1 days after sepsis onset) are elevated in patients with sepsis. Copy numbers of **(A)** mitochondrially encoded NADH-ubiquinone oxidoreductase chain 1 (*mt-ND1*), **(B)** mitochondrially encoded cytochrome C oxidase subunit III (*mt-CO3*) and **(C)** nuclear ribosomal protein S18 (*n-Rps18*) were determined by quantitative real-time PCR in plasma from healthy controls (n=81) and patients with sepsis (n=150) at T1. The y-axis is log10-transformed. The data is displayed as violin plots (solid lines: median, dashed lines: first and third quartiles; *p < 0.05 Mann-Whitney *U* test).

At T1, copy numbers of *mt-ND1* (median [IQR]; sepsis 963 [444–2 890] *vs* controls 678 [242–1 313], p=0.0006) and *n-Rps18* (sepsis 1 477 [528–4 721] *vs* controls 48 [0–220], p < 0.0001), but not *mt-CO3* (sepsis 447 [167–1 450] *vs* controls 324 [170–739], p=0.1145), were elevated in patients with acute sepsis compared to controls (**Figures 2A–C**).

### 3.3 Levels of circulating mtDNA and nDNA decrease after the acute phase of sepsis

We hypothesized that cfDNA is increased only in the acute phase of sepsis, and normalizes in the long course, as organ damage subsides. To test this hypothesis, we studied the longitudinal course of cfDNA over T2, T4 and T5.

**Figure 3:**
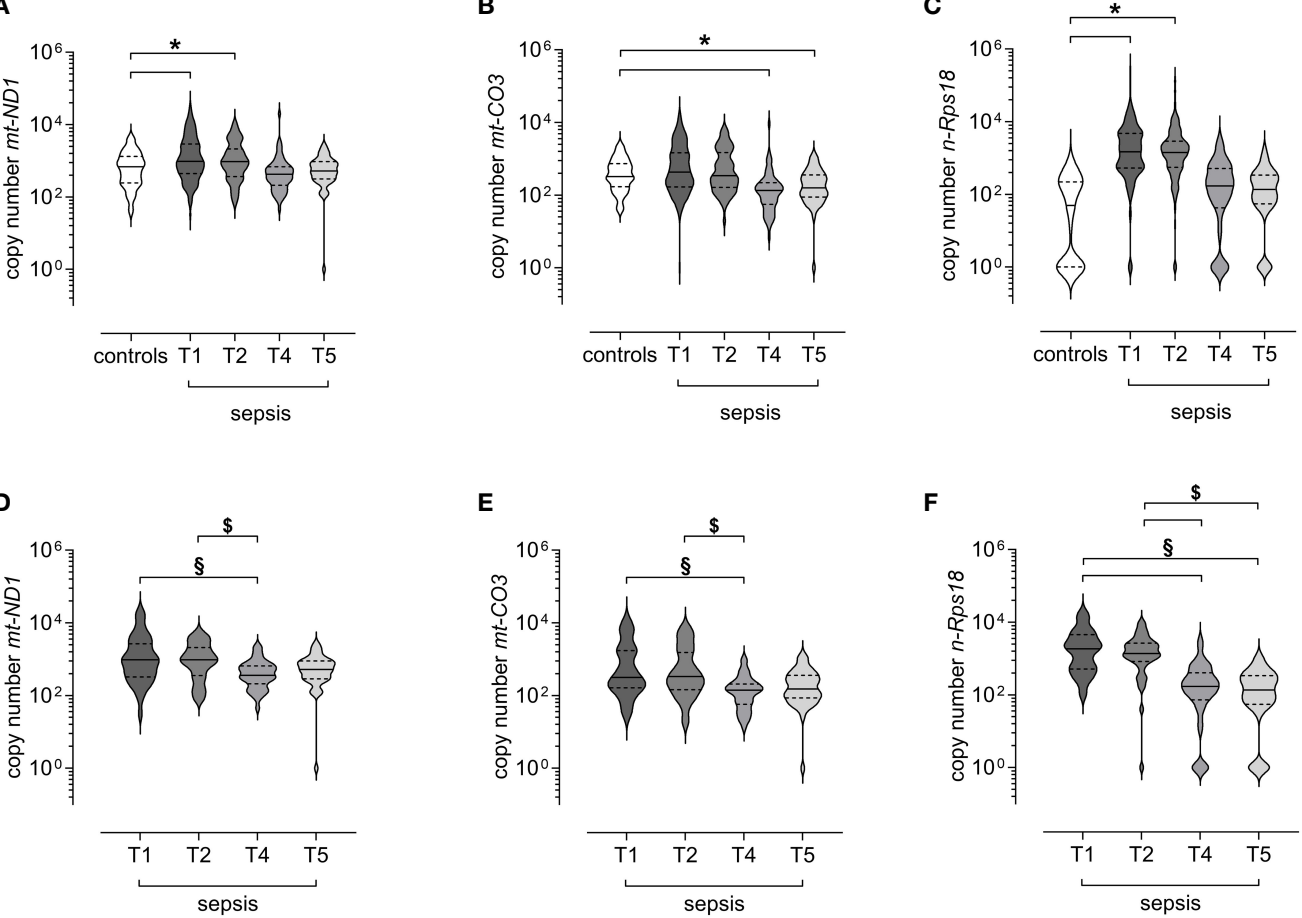
Levels of circulating mitochondrial DNA and nuclear DNA decrease after the acute phase of sepsis. Copy numbers of **(A)** mitochondrially encoded NADH-ubiquinone oxidoreductase chain 1 (*mt-ND1*), **(B)** mitochondrially encoded cytochrome C oxidase subunit III (*mt-CO3*), and **(C)** nuclear ribosomal protein S18 (*n-Rps18*) were determined by quantitative real-time PCR in plasma from healthy controls (n=81) and all available samples of patients with sepsis obtained at 3 ± 1 days (T1, n=150), 7 ± 1 days (T2, n=140), 6 ± 2 months (T4, n=65) and 12 ± 2 months (T5, n=45) after sepsis onset. Copy numbers of **(D)** *mt-ND1*, **(E)** *mt-CO3*, and **(F)** *n-Rps18* from survivors of sepsis with the complete long-term course up to 12 months after sepsis onset (n=41 patients with blood sampling for all study visits). The y-axis is log10-transformed. The data is displayed as violin plots (solid lines: median, dashed lines: first and third quartiles; *p < 0.05 versus controls in Dunn’s *post hoc* test for the Kruskal-Wallis-tests; §p < 0.05 *vs* T1, $p < 0.05 *vs* T2 in Dunn’s *post hoc* test for the Friedman tests).

First, we compared the long-term course in all patients with available data (varying patient numbers per study visit; **Figure 1**) to healthy controls. While copy numbers of *mt-ND1* and *n-Rps18* were significantly elevated at T1 and T2 compared to healthy controls, the copy numbers at T4 and T5 returned to control level (**Figures 3A, C**, **Supplementary Table 2**). In contrast, copy numbers of *mt-CO3* were comparable to control levels at T1 and T2 and significantly decreased at T4 and T5 compared to healthy controls (**Figure 3B**, **Supplementary Table 2**).

Second, we analyzed only survivors with the complete long-term course up to 12 months after sepsis onset (n=41 patients with blood sampling for all study visits). We observed no differences between T1 and T2 (**Figures 3D–F**, **Supplementary Table 2**). However, all three variables were significantly lower at T4 compared with T1 and T2 (**Figures 3D–F**, **Supplementary Table 2**). The copy number of *n-Rps18* was also significantly lower at T5 compared with T1 and T2 (**Figure 3F**, **Supplementary Table 2**). In conclusion, with the exception of *mt-CO3*, cfDNA is increased in the early stages of sepsis but decreases in the long-term after recovery from sepsis.

### 3.4 Levels of circulating mtDNA and nDNA predict 28-day mortality in sepsis

We hypothesize that circulating cfDNA levels reflect the degree of tissue damage and sepsis severity and may therefore be higher in non-survivors than in survivors of sepsis.

**Figure 4:**
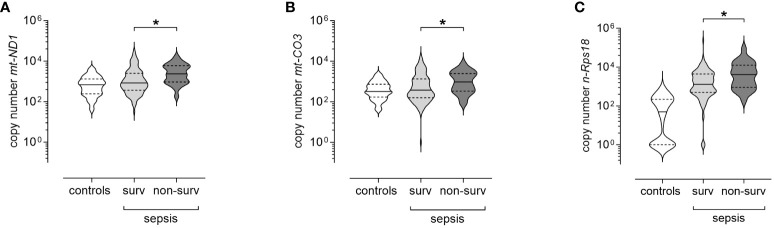
Levels of circulating mitochondrial DNA and nuclear DNA at T1 (3 ± 1 days after sepsis onset) are elevated in 28-day non-survivors of sepsis. Copy numbers of **(A)** mitochondrially encoded NADH-ubiquinone oxidoreductase chain 1 (*mt-ND1*), **(B)** mitochondrially encoded cytochrome C oxidase subunit III (*mt-CO3*) and **(C)** nuclear ribosomal protein S18 (*n-Rps18*) were determined by quantitative real-time PCR in plasma at T1 in 28-day survivors (surv, n=126) and non-survivors (non-surv, n=24). Healthy controls are shown for reference purposes (n=81). The y-axis is log10-transformed. The data is displayed as violin plots (solid lines: median, dashed lines: first and third quartiles; *p < 0.05, Mann-Whitney *U* test).

When stratified for 28-day mortality, we observed significantly higher copy numbers of *mt-ND1* (median [IQR]; non-survivors 2 342 [931–5 937] *vs* survivors 837 [361–2 506], p=0.002, **Figure 4A**), *mt-CO3* (non-survivors 959 [335–2 470] *vs* survivors 373 [156–1 336], p=0.028, **Figure 4B**) and *n-Rps18* (non-survivors 4 123 [919–12 437] *vs* survivors 1 294 [500–4 433], p=0.006, **Figure 4C**) in 28-day non-survivors (16%, n=24/150) compared with 28-day survivors. AKI is a known risk factor for mortality in patients with sepsis. In our study, 13 out of 44 patients requiring RRT died within 28 days. Of the 24 non-survivors, 21 either presented with or developed AKI during their ICU stay. Therefore, we performed binary logistic regression and ROC curve analysis to assess the predictive potential of SCr and the three cfDNA parameters for 28-day mortality.

**Figure 5:**
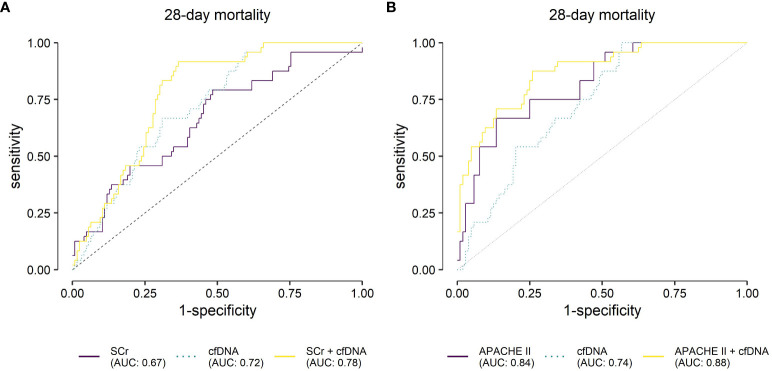
Receiver operating characteristic analysis for the predicted probabilities of the binary logistic regression models for 28-day mortality. **(A)** Evaluation of serum creatinine (SCr), mitochondrially encoded NADH-ubiquinone oxidoreductase chain 1 (*mt-ND1*), mitochondrially encoded cytochrome C oxidase subunit III (*mt-CO3*) and nuclear ribosomal protein S18 (*n-Rps18*) at T1 (3 ± 1 days after sepsis onset) and their combination as predictive biomarkers for 28-day mortality in patients with sepsis (n=24/150 non-survivors). Cell-free (cfDNA) comprises nuclear and mitochondrial DNA. **(B)** Evaluation of APACHE II, *mt-ND1*, *mt-CO3*, *n-Rps18* at T1 and their combination as predictive biomarkers for 28-day mortality in patients with sepsis (n=24/128 non-survivors) in patients with available Acute Physiology and Chronic Health Evaluation II scores (APACHE II). AUC, area under the curve.

Serum creatinine alone was a poor predictor of 28-day mortality (AUC=0.67, **Figure 5A** and **Table 2**). However, the three cfDNA parameters non-significantly improved the prediction (AUC=0.72, **Figure 5A** and **Table 2**, DeLong Test: one-sided test of superiority p=0.245), while the combination of all four parameters yielded the highest predictive value for 28-day mortality (AUC=0.78, **Figure 5A** and **Table 2**, DeLong Test *vs* SCr alone: one-sided test of superiority p<0.01).

**Table 2:** Logistic regression and Receiver Operating Characteristic (ROC) analysis for 28-day mortality.

		Logistic regression		ROC analysis			
Model	Variable	OR (95% CI)	p	AUC (95% CI)	Cut-off	sensitivity	specificity
**SCr**							
**A**	SCr^a^	1.73 (1.16–2.58)	**0.007**	0.67 (0.55–0.79)	0.12	79.2	52.4
**B**	*mt-ND1*	5.36 (0.61–47.14)	0.130	0.72 (0.63–0.82)	0.10	95.8	41.3
	*mt-CO3*	0.42 (0.08–2.22)	0.306				
	*n-Rps18*	1.77 (0.79–3.97)	0.168				
**C**	SCr^a^	1.69 (1.09–2.60)	**0.018**	0.78 (0.70–0.86)	0.14	91.7	64.3
	*mt-ND1*	6.79 (0.74–62.37)	0.091				
	*mt-CO3*	0.35 (0.06–1.95)	0.231				
	*n-Rps18*	1.50 (0.68–3.30)	0.313				
**APACHE II**							
**A**	APACHE II^a^	4.67 (2.40–9.08)	**<0.001**	0.84 (0.75–0.92)	0.35	66.7	86.5
**B**	*mt-ND1*	6.47 (0.81–51.80)	0.079	0.74 (0.65–0.83)	0.11	100.0	44.2
	*mt-CO3*	0.34 (0.07–1.66)	0.181				
	*n-Rps18*	1.82 (0.82–4.03)	0.143				
**C**	APACHE II^a^	5.77 (2.60–12.77)	**<0.001**	0.88 (0.80–0.95)	0.16	87.5	75.0
	*mt-ND1*	26.81 (2.22–323.77)	**0.010**				
	*mt-CO3*	0.09 (0.01–0.61)	**0.013**				
	*n-Rps18*	1.26 (0.52–3.03)	0.608				

APACHE II, Acute Physiology and Chronic Health Evaluation II; mt-ND1, mitochondrially encoded NADH-ubiquinone oxidoreductase chain 1; mt-CO3, mitochondrially encoded cytochrome C oxidase subunit III; n-Rps18, nuclear ribosomal protein S18; AUC, area under the curve; OR, odds ratio; 95% CI, 95% confidence interval; ^a^ values of SCr and APACHE II were z-standardized before modeling (mean=0, standard deviation=1); p, p value of the OR (p<0.05 are printed in bold).

Results are reported for the models with serum creatinine (SCr, n=150; n=24/150 non-survivors) and APACHE II scores at T1 (n=128 patients; n=24/128 non-survivors) as main covariable.

Next, we analyzed the predictive value of cfDNA when compared to APACHE II, an established and routinely used clinical score. Analogous to our one-point analysis of cfDNA at T1, APACHE II does not need sequential measurements during ICU stay, but can reflect severity of disease and predict mortality in critically ill patients (33, 34). In our cohort, APACHE II scores at T1 were not available for 22 patients due to multiple missing values. We therefore excluded these patients from the analysis. Interestingly, APACHE II scores only correlated poorly with cfDNA (Spearman’s ρ=0.251, p=0.004 for *mt-ND1*, ρ=0.268, p=0.002 for *mt-CO3* and ρ=0.272, p=0.002 for *n-Rps18*) at T1. As expected, we found that APACHE II predicts 28-day mortality (AUC=0.84, **Figure 5B** and **Table 2**). The model with cfDNA performed poorer (AUC=0.74, DeLong Test: one-sided test of superiority p=0.948). Nonetheless, the combined model performed best (AUC=0.88, **Figure 5B** and **Table 2**, DeLong Test *vs* APACHE II alone: one-sided test of superiority p=0.026). In the combined model, *mt-ND1* and *mt-CO3* significantly contributed to the prediction of 28-day mortality (**Table 2**).

### 3.5 Levels of circulating mtDNA and nDNA are elevated in patients with sepsis-associated AKI

We next evaluated whether cfDNA can be used as a marker for SA-AKI. Even though attention has been lately shifted to the role of mtDNA in AKI, studies analyzing the relationship between AKI and nDNA are scarce.

**Figure 6:**
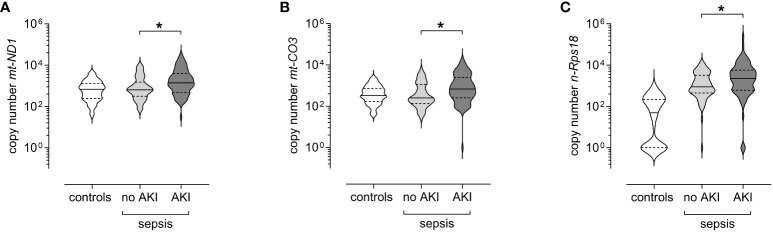
Levels of circulating mitochondrial DNA and nuclear DNA at T1 (3 ± 1 days after sepsis onset) are elevated in patients with sepsis-associated AKI. Copy numbers of **(A)** mitochondrially encoded NADH-ubiquinone oxidoreductase chain 1 (*mt-ND1*), **(B)** mitochondrially encoded cytochrome C oxidase subunit III (*mt-CO3*), and **(C)** nuclear ribosomal protein S18 (*n-Rps18*) were determined by quantitative real-time PCR in plasma from patients with sepsis and AKI (AKI, n=82) or without AKI (no AKI, n=68) obtained at T1. Healthy controls are shown for reference purposes (n=81). The y-axis is log10-transformed. The data is displayed as violin plots (solid lines: median, dashed lines: first and third quartiles; *p < 0.05 Mann-Whitney *U* test).

In our cohort, SA-AKI was diagnosed in 54.7% of patients. At T1, patients with SA-AKI showed significantly elevated levels of *mt-ND1* (median [IQR]; with SA-AKI 1393 [483–4 004] *vs* without SA-AKI 637 [313–1 523], p<0.001), *mt-CO3* (with SA-AKI 686 [255–2 507] *vs* without SA-AKI 249 [133–1 158], p<0.001) and *n-Rps18* (with SA-AKI 2 235 [602–5 773] *vs* without SA-AKI 890 [449–3 222], p=0.006) (**Figures 6A–C**).

### 3.6 Levels of circulating mtDNA and nDNA are elevated in patients with sepsis requiring RRT

Having found that cfDNA is increased in SA-AKI, we next investigated whether levels of cfDNA scale with the severity of SA-AKI. As a clinically relevant stage, we chose the need of RRT (equivalent to AKI stage 3) up to day 28 as a comparator.

**Figure 7:**
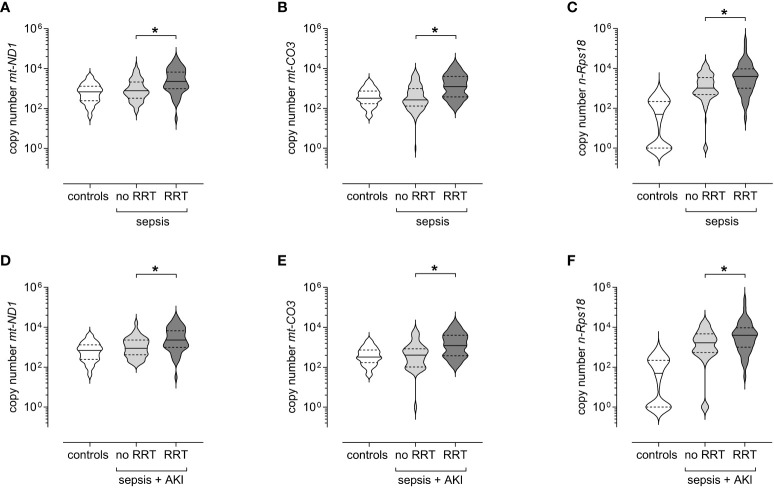
Circulating levels of mitochondrial DNA and nuclear DNA at T1 (3 ± 1 days after sepsis onset) are elevated in patients with sepsis requiring renal replacement therapy (RRT) up to day 28. Copy numbers of **(A)** mitochondrially encoded NADH-ubiquinone oxidoreductase chain 1 (*mt-ND1*), **(B)** mitochondrially encoded cytochrome C oxidase subunit III (*mt-CO3*) and **(C)** nuclear ribosomal protein S18 (*n-Rps18*) were determined by quantitative real-time PCR in plasma obtained at T1 from patients with sepsis who required RRT up to day 28 (n=44) and patients with sepsis who did not (n=106). Healthy controls are shown for reference purposes (n=81). Copy numbers of **(D)** *mt-ND1*, **(E)** *mt-CO3* and **(F)** *n-Rps18* were determined by quantitative real-time PCR in plasma obtained at T1 from patients with sepsis and AKI who required RRT up to day 28 (n=44) and patients with sepsis and AKI who did not (n=38). Healthy controls are shown for reference purposes (n=81). The y-axis is log10-transformed. The data is displayed as violin plots (solid lines: median, dashed lines: first and third quartiles; *p < 0.05 Mann-Whitney *U* test).

Forty-four of the patients with sepsis required RRT during their ICU stay. We observed elevated levels of *mt-ND1* (median [IQR]; with RRT 2 273 [973–6 661] *vs* without RRT 775 [328–2 125], p<0.001), *mt-CO3* (with RRT 1 211 [376–4 064] *vs* without RRT 267 [128–977], p<0.001), and *n-Rps18* (with RRT 3 939 [1 003–9 494] *vs* without RRT 1 028 [489–3 474], p<0.001) in patients with RRT compared to patients without RRT (**Figures 7A–C**).

All patients requiring RRT suffered from AKI, however, not all patients with AKI required RRT. Therefore, we analyzed patients with sepsis and AKI with and without RRT. When looking only at patients with SA-AKI, those receiving RRT also had higher levels of cfDNA compared to patients with SA-AKI without RRT, as follows (**Figures 7D–F**): *mt-ND1* (median [IQR]; with RRT 2 273 [973–6 661] *vs* without RRT 882 [415–2 280], p=0.003), *mt-CO3* (with RRT 1 211 [376–4 064] *vs* without RRT 401 [101–845], p<0.001), *n-Rps18* (with RRT 3 939 [1 003–9 494] *vs* without RRT 1 672 [533–4 646], p=0.036).

### 3.7 Levels of circulating *mt-CO3* improve the predictive value of serum creatinine for RRT

The increase of cfDNA in sepsis may not specifically reflect SA-AKI, but rather other confounders, such as the severity of sepsis. To better quantify this relationship, we performed binary logistic regression and ROC curve analysis to compare the potential of mt- and nDNA as a biomarker for RRT and compared these to the currently universally used SCr.

**Figure 8:**
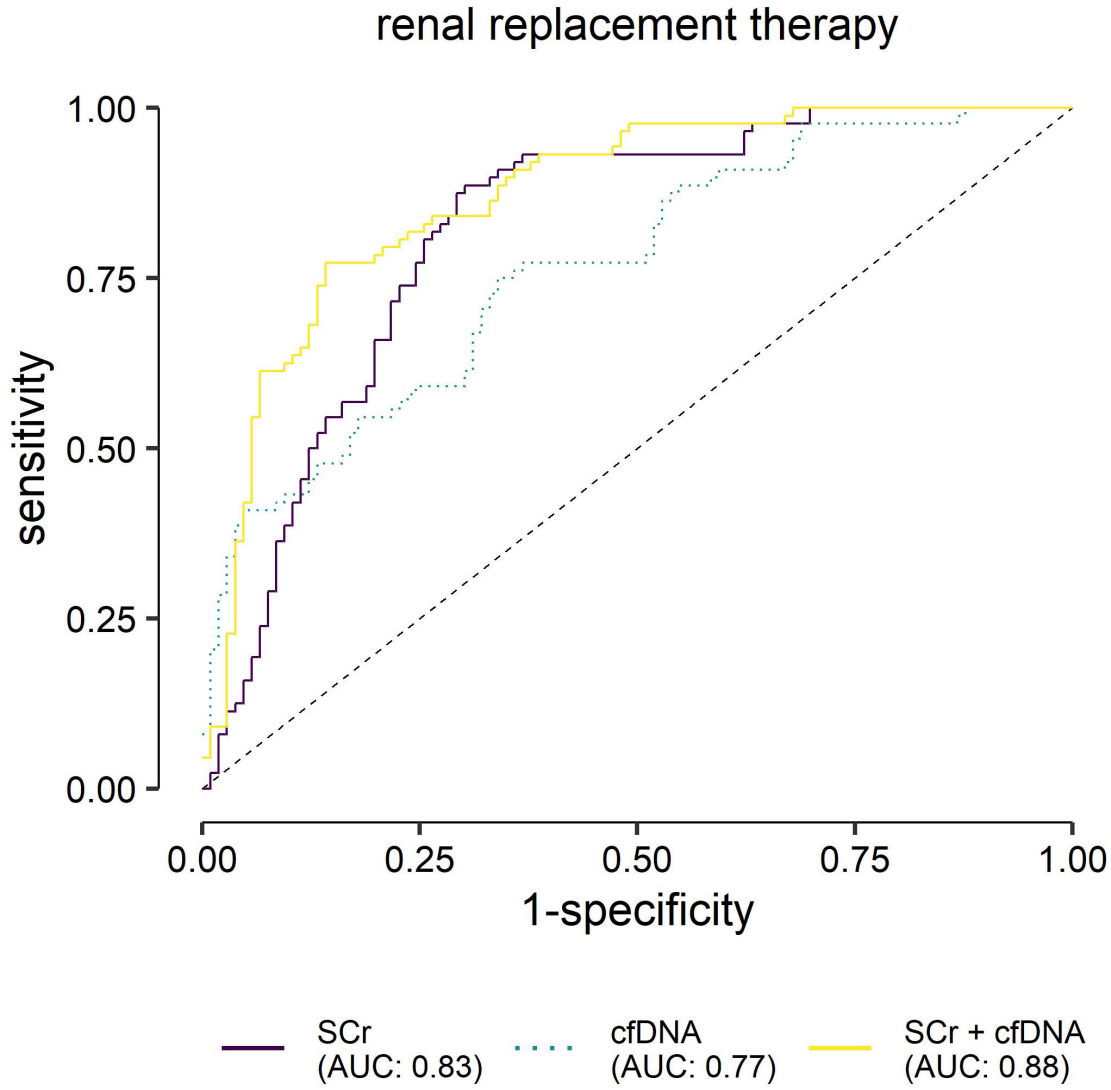
Receiver operating characteristic analysis for the predicted probabilities of the binary logistic regression models for renal replacement therapy (RRT). Evaluation of serum creatinine (SCr), mitochondrially encoded NADH-ubiquinone oxidoreductase chain 1 (*mt-ND1*), mitochondrially encoded cytochrome C oxidase subunit III (*mt-CO3*) and nuclear ribosomal protein S18 (*n-Rps18*) at T1 (3 ± 1 days after sepsis onset), and their combination as biomarkers for RRT (n=44/150) in patients with sepsis. Cell-free (cfDNA) comprises nuclear and mitochondrial DNA.

The combination of *mt-ND1*, *mt-CO3* and *n-Rps18* yielded a lower diagnostic performance (AUC=0.77, DeLong Test *vs* SCr alone: one-sided test of superiority p=0.845) than SCr (AUC=0.83, **Figure 8** and **Table 3**). However, combining SCr with the three cfDNA variables resulted in a significantly improved diagnostic performance (AUC=0.88, **Table 3**, DeLong Test *vs* SCr alone: one-sided test of superiority p=0.047). The composite model C including SCr and the DNA parameters shows slight improvements in specificity (**Table 3**). Interestingly, next to SCr, only *mt-CO3* contributed significantly to the prediction of RRT (**Table 3**).

**Table 3:** Logistic regression and Receiver Operating Characteristic (ROC) analysis for renal replacement therapy up to day 28 (n=150 patients).

		Logistic regression		ROC analysis			
Model	Variable	OR (95% CI)	p	AUC (95% CI)	Cut-off	sensitivity	specificity
**A**	SCr	2.97 (1.95–4.52)	**<0.001**	0.83 (0.76–0.89)	0.21	88.6	70.8
**B**	*mt-ND1*	0.29 (0.06–1.41)	0.125	0.77 (0.69–0.85)	0.27	75.0	67.0
	*mt-CO3*	9.87 (2.34–41.58)	**0.002**				
	*n-Rps18*	2.21 (1.04–4.73)	**0.040**				
**C**	SCr	3.41 (2.08–5.57)	**<0.001**	0.88 (0.82–0.93)	0.39	77.3	86.8
	*mt-ND1*	0.35 (0.06–2.22)	0.266				
	*mt-CO3*	13.3 (2.46–71.97)	**0.003**				
	*n-Rps18*	1.58 (0.75–3.32)	0.226				

SCr, serum creatinine (z-standardized before modeling; mean=0, standard deviation=1); mt-ND1, mitochondrially encoded NADH-ubiquinone oxidoreductase chain 1; mt-CO3, mitochondrially encoded cytochrome C oxidase subunit III; n-Rps18, nuclear ribosomal protein S18; AUC, area under the curve; OR, odds ratio; 95% CI, 95% confidence interval; p, p value of the OR (p<0.05 are printed in bold).

## 4 Discussion

In this study, the applicability of liquid biopsies in the form of circulating cfDNA was investigated in a mixed population of 150 surgical and non-surgical patients with sepsis. We measured two genes of mitochondrial cfDNA, *mt-ND1* (within the mitochondrial complex I) and *mt-CO3* (within the mitochondrial complex IV), as well as a nuclear gene of cfDNA, *n-Rps18*. Mitochondrial DNA has been previously investigated in patients with kidney damage (35–37), while nuclear cfDNA has been studied for possible associations with tissue and organ damage in sepsis and septic shock (22, 38). Our analysis investigated the trajectory of cfDNA, as well as the predictive and prognostic value of cfDNA for 28-day mortality in patients with sepsis, its association with AKI and its role in predicting the need for RRT in patients with AKI.

### 4.1 *mt-ND1* and nDNA are increased in patients with sepsis and return to control values during long-term recovery

Similar to previous data (22, 39, 40), we found that, compared to healthy controls, patients with sepsis have significantly higher levels of cfDNA in the acute phase of sepsis. Nuclear DNA in particular, and to a lesser extent mtDNA, showed significantly increased levels in the acute phase of sepsis (in our study defined as T1, 3 ± 1 days after onset of sepsis and as T2, 7 ± 1 days) as a possible reflection of acute tissue damage. Other studies performed serial measurements of cfDNA and found increased levels of nDNA up to 28 days after the onset of sepsis or septic shock (22). To the authors’ knowledge, ours is the first study to look at the long-term dynamics of cfDNA up to 6 and 12 months after sepsis. We found in our cohort that the initially high levels of cfDNA decrease over time. The greatest change was seen with nDNA, raising the question whether nDNA is more relevant in the acute phase of sepsis compared with mtDNA. Hawkins et al. found that nDNA, but not mtDNA, was associated with adverse outcomes, and nDNA at 12 hours (but not at 1, 4, or 7 days) after sepsis onset was a predictor of 28-day mortality (along APACHE II and SOFA scores) (40). Such a narrow time window casts doubt over the reliability and clinical utility of nDNA as a predictor of adverse events in patients with sepsis. Timmermans et al. also found elevated nDNA levels in patients with septic shock that correlated weakly with the inflammatory response and markers of organ damage, but reported no clinical outcomes (22).

### 4.2 Cell-free DNA is increased in non-survivors of sepsis and can improve the predictive value of APACHE II for mortality

We found that cfDNA can contribute to the prediction of 28-day mortality, both alone and by increasing the AUC of APACHE II. Our results confirm the already existing body of work that showed that cfDNA has the power to improve risk prediction models (41–43) which could lend it an important role in therapy decisions in the ICU. An important observation is that only one determination of cfDNA (at T1) was sufficient for this improvement, hence such determinations may be economically viable. As discussed above, despite a stronger increase in nDNA during early sepsis, mtDNA had a stronger predictive value both for RRT (*mt-CO3*), as well as for mortality (*mt-ND1* and *mt-CO3*). This raises the question whether mtDNA may act as both markers and mediators of secondary tissue damage. Indeed, mtDNA has been proposed to mediate inflammatory pathways by acting as a potent damage-associated molecular patterns (DAMPs) (43, 44). Future studies will need to look at the individual trajectories of mitochondrial cfDNA and nuclear cfDNA to examine specific correlations with individual outcomes such as AKI or all-cause sepsis mortality.

### 4.3 Cell-free DNA is increased in SA-AKI, and can improve the predictive value of serum creatinine for RRT

Diagnosing AKI in the context of sepsis remains a difficult task. The KDIGO definition is based on SCr and urine output (2, 45). However, these parameters reflect kidney function and have limited value in detecting kidney injury (8, 46), which is the paramount characteristic of AKI, as recognized in its name. One of the biggest conundrums concerns the baseline creatinine value, which should be considered when calibrating the acute decrease in kidney function. In many patients such values are missing, which leads to delays in diagnosis. The interest is shifting toward finding biomarkers which could describe renal injury. In the investigated cohort, SA-AKI could be diagnosed in around 55% of patients, an incidence comparable with that presented in other studies (47–49). In our study, cfDNA was not only increased in SA-AKI, but it also scaled with the intensity of renal damage, which was mirrored by the need for RRT. Due to the clinical impact of RRT and to the fact that it can directly affect the outcome in patients with sepsis, we focused our attention further on the relationship between cfDNA and the need for RRT. According to KDIGO, the need for RRT translates to a stage 3 AKI. Interestingly, despite the fast turnover of cfDNA, the values determined at T1 (3 days after sepsis onset) still carried prognostic value regarding SA-AKI stage 3 (equivalent to RRT) during the first 28 days of ICU treatment. While some animal studies found a relationship between cfDNA and AKI (37), data from human studies are still heterogeneous and, at times, contradictory. Similar to our data, but in a smaller patient population, Clementi et al. could also correlate levels of cfDNA to development of AKI and the need for RRT in patients with sepsis (50). Intriguingly, other studies could correlate cfDNA levels in urine with AKI, but not plasmatic cfDNA (51). In our study, cfDNA showed a promising predictive capability for RRT (AUC 0.77) and could significantly improve the prediction capacity of SCr for the same event, with *mt-CO3* showing the most substantial contribution. With the addition of mtDNA to the model, AUC reached 0.88. From a clinical standpoint, this finding may be of value since it only requires measurement of *mt-CO3* at one time point and does not require a baseline value before the onset of sepsis, as is the case with creatinine.

## 5 Limitations and strengths

One of the major strengths of the present study is that we were able to describe the long-term trajectory of cfDNA in sepsis survivors. This study followed patients up to 12 months after the onset of sepsis and provides valuable data on the dynamics of the mitochondrial and nuclear DNA over time.

Our study also has some limitations. An important limitation, given the rapid elimination rate of cfDNA, is the lack of serial measurements during the acute phase of sepsis. Determining the temporal relationship between cfDNA levels and the development of SA-AKI would likely have improved the prognostic value of our markers.

Another limitation, similar to many other studies investigating SA-AKI, resides in the difficulty of diagnosing AKI based on the current KDIGO criteria in patients where SCr over the last 7 days is missing. We conscientiously reviewed the patients’ electronic medical records and were thus able to complete any missing information. Even though this method is in line with the current literature (52), we cannot exclude with certainty that we did not under- or overestimated the incidence of AKI or the staging of the injury, a pitfall common with other similar investigations (53).

In future studies, it would be interesting to address whether nDNA and mtDNA levels differ depending on the clinical phenotype of SA-AKI. This should take into account the variety of pathophysiological mechanisms that seem to play a role in triggering damage in SA-AKI. In addition, it would be interesting to gain insights into the relationship between circulating cfDNA and urinary DNA in order to investigate which markers and measurements provide more reliable data. In addition, it would be interesting to gain insights into the relationship between circulating cfDNA and urinary DNA to investigate which markers and measurements provide more reliable data, as recent studies have shown that urinary mtDNA levels predict the severity of AKI (54) and sepsis-associated AKI (55).

The lack of diversity as well as the relatively modest size of our patient cohort must also be addressed, as this may limit the generalizability of the results. Future analysis could attempt to investigate more diverse and larger populations. In particular, the predictive models should be validated in independent cohorts in order to clarify the prognostic relevance cfDNA in the clinical setting.

## 6 Conclusion

In conclusion, our study provides important data on the clinical value of Cell-free DNA, represented here by *mt-ND1*, *mt-CO3* and *n-Rps18* in patients with sepsis. Cell-free DNA parameters alone may play a role in predicting mortality and morbidity in patients with sepsis. However, they could be much more valuable as part of composite scores. Of considerable advantage is the predictive value of such markers even with a single measurement at the onset of sepsis, and the fact that no baseline measurement is required. Especially the latter could help to accelerate the diagnosis of sepsis complications such as SA-AKI in patients with previous renal damage. Considering that our data show that cfDNA levels decrease after the initial insult, future studies could investigate cfDNA as a “memoryless” marker.

## Data availability statement

The original contributions presented in the study are included in the article/**Supplementary Material**, further inquiries can be directed to the corresponding author/s.

## Ethics statement

The study was approved by the Ethics Committee of the Friedrich Schiller University Jena. The study was conducted in accordance with the local legislation and institutional requirements. Every participant, legal representative or proxy gave their signed written informed consent or, if none of the situations applied, an independent medical doctor was consulted for preliminary consent.

## Author contributions

SD: Conceptualization, Data curation, Formal analysis, Investigation, Methodology, Visualization, Writing – original draft, Writing – review & editing. I-AC: Data curation, Formal analysis, Methodology, Writing – original draft, Writing – review & editing. PB: Formal analysis, Methodology, Visualization, Writing – review & editing. MA: Data curation, Investigation, Writing – review & editing. SK: Formal analysis, Visualization, Writing – review & editing. SMC: Conceptualization, Funding acquisition, Methodology, Project administration, Resources, Visualization, Writing – original draft, Writing – review & editing.
