# Supplementary material for: Cell-free DNA in patients with sepsis: long term trajectory and association with 28-day mortality and sepsis-associated acute kidney injury
Source: Front Immunol. 2024 May 13;15:1382003. doi: 10.3389/fimmu.2024.1382003 (PMC11128621; doi:10.3389/fimmu.2024.1382003)
Supplement: Supplementary file 1 [file DataSheet_1.docx]

Supplementary Material

Cell-free DNA in patients with sepsis: long term trajectory and association with 28-day mortality and sepsis-associated acute kidney injury

**Sophie Dennhardt^1,2,§^, Iuliana-Andreea Ceanga^1,2,§^, Philipp Baumbach^1,2,§^; Mona Amiratashani^1,2^, Sarah Kröller^1,2^; Sina M. Coldewey^1,2,3,*^**

^1^Department of Anesthesiology and Intensive Care Medicine, Jena University Hospital, Friedrich

Schiller University Jena, Jena, Germany,

^2^Septomics Research Centre, Jena University Hospital, Friedrich Schiller University Jena, Jena, Germany

^3^Center for Sepsis Control and Care, Jena University Hospital; Jena, Germany

^§^Authors contributed equally.

*** Correspondence:**

Prof. Sina M. Coldewey, MD PhD

Department of Anaesthesiology and Intensive Care Medicine

Jena University Hospital

Jena, Germany

[sina.coldewey@med.uni-jena.de](mailto:sina.coldewey@med.uni-jena.de)

# Supplementary Tables

**Supplementary Table 1**: **Collinearity analysis for the potential biomarkers of RRT and 28-day mortality.** Variance inflation factors (VIF) were calculated in SPSS and are under the cut-off value of VIF = 10 indicating no significant collinearity between parameters.

| marker | VIF (RRT) | VIF (28-day mortality) |
| --- | --- | --- |
| *mt-ND1* | 6.168 | 5.948 |
| *mt-CO3* | 5.963 | 5.840 |
| *nc-Rps18* | 1.099 | 1.096 |
| SCr | 1.024 | 1.101 |
| APACHE II | n/a | 1.133 |

**Supplementary Table 2: Median, IQR and p-values for the long-course of circulating mitochondrial and nuclear DNA.**

| **Figure 3: Panel A-C** | | | |
| --- | --- | --- | --- |
|  | *mt-ND1* | *mt-CO3* | *nc-Rps18* |
| median controls | 678 | 324 | 48 |
| IQR controls | 242 - 1313 | 170 - 739 | 0 - 220 |
| median T1 | 963 | 428 | 1477 |
| IQR T1 | 444 - 2890 | 167 - 1450 | 528 - 4721 |
| p-value T1 vs. controls | 0.0026 | 0.7615 | <0.0001 |
| median T2 | 950 | 345 | 1411 |
| IQR T2 | 364 - 2113 | 163 - 1471 | 556 - 2902 |
| p-value T2 vs. controls | 0.0216 | >0.9999 | <0.0001 |
| median T4 | 421 | 134 | 170 |
| IQR T4 | 210 - 680 | 55 - 220 | 42 - 513 |
| p-value T4 vs. controls | 0.1831 | <0.0001 | 0.0902 |
| median T5 | 521 | 156 | 135 |
| IQR T5 | 315 - 952 | 87 - 359 | 54 - 338 |
| p-value T5 vs. controls | >0.9999 | 0.0067 | 0.6828 |
|  | | | |
| **Figure 3: Panel D-F** | | | |
|  | *mt-ND1* | *mt-CO3* | *nc-Rps18* |
| median T1 | 960 | 314 | 1840 |
| IQR T1 | 321 - 2676 | 162 - 1708 | 510 - 4544 |
| p-value T1 vs. T2 | >0.9999 | >0.9999 | >0.9999 |
| p-value T1 vs. T4 | 0.0165 | 0.0007 | <0.0001 |
| p-value T1 vs. T5 | 0.4346 | 0.0787 | <0.0001 |
| median T2 | 966 | 330 | 1370 |
| IQR T2 | 354 - 2098 | 143 - 1538 | 829 - 2666 |
| p-value T2 vs. T4 | 0.0093 | 0.0007 | <0.0001 |
| p-value T2 vs. T5 | 0.2948 | 0.0787 | <0.0001 |
| median T4 | 359 | 140 | 170 |
| IQR T4 | 210 - 653 | 56 - 206 | 72 - 405 |
| median T5 | 521 | 150 | 135 |
| IQR T5 | 285 - 893 | 85 - 359 | 54 - 338 |
| p-value T4 vs. T5 | >0.9999 | >0.9999 | >0.9999 |
